# Effect of rock properties on wear and cutting performance of multi blade circular saw with iron based multi-layer diamond segments

**DOI:** 10.1038/s41598-024-54625-5

**Published:** 2024-02-26

**Authors:** Sohan Singh Rajpurohit, Yewuhalashet Fissha, Rabindra Kumar Sinha, Mujahid Ali, Hajime Ikeda, Wade Ghribi, Taoufik Najeh, Yaser Gamil, Youhei Kawamura

**Affiliations:** 1 Department of Mining Engineering, Indian Institute of Technology (Indian School of Mines), Dhanbad, 826004 India; 2 Department of Geosciences, Geotechnology, and Materials Engineering for Resources, Graduate School of International Resource Sciences, Akita University, Akita, Japan; 3 Department of Mining Engineering, Aksum University, 7080 Aksum, Tigray Ethiopia; 4 Department of Transport Systems, Traffic Engineering and Logistics, Faculty of Transport and Aviation Engineering, Silesian University of Technology, Krasińskiego 8 Street, 40-019 Katowice, Poland; 5 Department of Computer Engineering, College of Computer Science, King Khalid University, Abha, Saudi Arabia; 6 Operation and Maintenance, Operation, Maintenance and Acoustics, Department of Civil, Environmental and Natural Resources Engineering, Luleå University of Technology, Luleå, Sweden; 7 Department of Civil Engineering, School of Engineering, Monash University Malaysia, Jalan Lagoon Selatan, 47500 Bandar Sunway, Selangor Malaysia; 8 Faculty of Engineering, Hokkaido University, Kita 8, Nishi 5, Kita-ku, Sapporo, 0608628 Japan

**Keywords:** Circular saw, Wear rate, Tribology, Granite, Rock property, Multi-layer diamond segment, Quarry, Engineering, Materials science

## Abstract

This study is an attempt for comprehensive, combining experimental data with advanced analytical techniques and machine learning for a thorough understanding of the factors influencing the wear and cutting performance of multi-blade diamond disc cutters on granite blocks. A series of sawing experiments were performed to evaluate the wear and cutting performance of multi blade diamond disc cutters with varying diameters in the processing of large-sized granite blocks. The multi-layer diamond segments comprising the Iron (Fe) based metal matrix were brazed on the sawing blades. The segment’s wear was studied through micrographs and data obtained from the Field Emission Scanning Electron Microscopy (FESEM) and Energy Dispersive X-ray (EDS). Granite rock samples of nine varieties were tested in the laboratory to determine the quantitative rock parameters. The contribution of individual rock parameters and their combined effects on wear and cutting performance of multi blade saw were correlated using statistical machine learning methods. Moreover, predictive models were developed to estimate the wear and cutting rate based on the most significant rock properties. The point load strength index, uniaxial compressive strength, and deformability, Cerchar abrasivity index, and Cerchar hardness index were found to be the significant variables affecting the sawing performance.

## Introduction

Large diameter segmented circular saw blades are predominantly used in the sawing of dimension stones^1,2^. Efficient cutting operations, the accuracy of the cut, high production capability, operability in hard stone such as granite are the main factors for its extensive usage in stone processing plants^3,4^. With the rising demand for processed natural stone slabs and tiles in the global natural building market, the focus of the tool production industry is shifting towards optimizing the sawing operations^5^. In order to achieve a higher production rate, the number of circular saw blades per machine has been gradually increased^6^. Moreover, the conventional diamond segments have been replaced by Iron based multi-layer diamond segments to improve the cutting and wear performance of saw^7–9^.

Cutting rate and wear rate are system dependent tribological parameters^10,11^. The multi objective function of the stone processing project is to optimize the cutting and wear rate^12–14^. The main limiting constraints are the rock characteristics, tool characteristics, and machine capacity^1,15^. Therefore, it is crucial for diamond tool manufacturers and processing plant engineers to understand the response of the target rock workpiece and its influencing parameters before selecting appropriate rock sawing tools^16–18^. After the tool selection, operational parameters can be optimized to maximize the feasible production performance^19–21^.

In this view, numerous research studies have been performed to investigate circular saw performance in terms of rock properties and operational parameters (Table 1). Most of them are limited to single disc cutters in conjunction with the conventional diamond segments. However, some experimental studies have been reported related to multi-blade saw performance for carbonate rocks^22^ and segment wear characteristics^23^. On top of this different researchers like^24–26^ have integrated the application of predictive models such as ANN, regression analysis, and statistical analysis in to their study. (Aydin et al.^25^) uses ANN and other regression models for predicting the diamond sawblades in rock sawing, especially for granite rock. In similar fashion (Karakurt et al.^26,27^) introduces an experimental and statistical study on noise level prediction which is generated during of rock sawing by circular diamond sawblades. He found that increasing of traverse speed, peripheral speed, and cutting depth result in an increase in noise levels during the cutting process.

**Table 1:** Summary of the previous research studies related to diamond disc cutter sawing performance prediction.

O/P	Rock properties used in the prediction models	Rock type	No. of samples	Method for prediction	Researcher(s)
CR	Gs, Qc	Swedish Granite	5	Empirical	^29^
CR	Gs, Qc, SH, Abrasion	Granite	6	Empirical	^30^
WR	SH, CAI	Granite, Marble	6	Empirical	^31^
WR	Mean grain size, CI,SH, MH	Granite	8	MLR	^32^
WR	UCS, Abrasivity, SH, Qc, Gs	Granite	7	Fuzzy ranking	^33^
WR	UCS, BTS, PLSI, SH, CI, LA, BA	Andesite	28	MLR	^16^
CR	C, ϕ, UCS, BTS, SHH, PLSI, IS, LA, V_p_	Carbonate rocks	13	Curvilinear reg	^34^
CR	Rock Brittleness	Carbonate	8	Log linear reg	^35^
CR	UCS, BS, Abrasivity	Carbonate	14	SLR, MLR	^15^
WR	UCS, BS, Abrasivity	Carbonate	14	SLR, MLR	^36^
CR	Qc, Vickers microhardness	Granite	10	SLR	^37^
CR	SS	Carbonate	13	ANN	^38^
CR	UCS, BTS, SHH, PLSI, IS, LA, Vp	Andesite	8	SLR	^12^
WR	UCS, BTS, BS, SHH, SSH, CAI, Density,Porosity, GS, Qc,	Granite	6	Subset regression	^39^
CR	Indentation Hardness (based on PLSI)	Carbonate	8	SLR	^40^
WR	UCS, BTS SH, Density, WA, Porosity, Qc, Gs	Granite	9	Subset MLR	^21^
CR	SH, SHH, Gs	Marble	5	SLR, MLR	^20^
WR	MH, Vickers Hardness, Rosiwal number	Granite	9	SLR, MLR	^21^
SE	UCS, BTS, BS, PLSI, SSH, SHH, V_p_, WA	Carbonate	6	Subset MLR	^41^
WR	SH, CI, BA, Brittleness	Marble	6	MLR	^14^
WR	UCS, BS, MH, Micro hardness, CAI, SH, MH, WA, Porosity, Vp, BS, SHH	Granite	9	MLR	^24^
CR	Density, Porosity, UCS, BTS, CAI	Carbonate	11	ANN	^1^
WR	Knoop hardness	Granite	10	SLR	^11^
CR	Density, UCS, BTS, CAI, SHH, SSH, GS	Carbonate	25	MLR, Non-linear Reg	^17^
CR	MH, UCS	Carbonate	7	SLR	^13^
CR	UCS, BTS, SF, MH, E, Gs, Qc	Granite, Marble	12	ANN	^42^
WR	KH, BTS, BS, CAI	Carbonate	13	SLR, MLR	^18^
TLI	SHH, BTS, Vp	Carbonate rocks	8	MLR, Non-linear Reg	^22^

*O/P* predcited output from the model, *CR* stone cutting rate, *WR* wear rate of diamond segments, *TLI* tool life index, *Gs* grain size of mineral, *Qc* quartz content (%), *UCS* uniaxial compressive strength, *BTS* tensile strength, *PLSI* point load strength index, *IS* impact strength, *BS* bending strength, *SS* shear strength, *C* cohesion, *ϕ* friction angle, *SHH* schmidt hammer rebound number hardness, *SH* shore hardness, *CI* cone indenter hardness, *MH* mohs hardness number, *CAI* cerchar abrasivity index, *A* bohme abrasion, *SFa* schimazek's **F**-value, *LA* los angles abrasion,, *WA* water absorption, *Vp* P-wave velocity, *ANN* artificial neural networks, *SLR* simple linear regression, *MLR* multiple linear regression*.*

Based on^27^ the fundamental significant operating variable affecting the cutting force in a granite rock is cutting depth. The surface roughness is primarily influenced by the peripheral speed and traverse speed, which are considered as the key operational factors. Moreover, the surface roughness of the rock is mostly attributed to its mineralogical qualities rather than its mechanical capabilities. The mean grain size of a rock is the most influential mineralogical property in determining surface roughness^28^.

The present study aims to evaluate the sawing performance of multi blade saw with multi-layer diamond segments. The main objective of this study is to investigate and quantify the influence of the governing rock properties significantly affecting the wear rate and cutting rate in sawing operations. A particular group of 9 different granites with varying physical, mechanical, petrographic, and aesthetic properties were cut in a commercial stone processing plant, and their rock properties were determined in the laboratory. The predictive models to estimate the wear and cutting rate based on the significant rock properties were obtained using statistical machine learning methods. The wear progression of diamond segments was investigated through the surface morphology of diamond grains and the bond matrix. The rest of the paper is organized as follows: Section 2 provides the Rock properties of granite, and the findings on multi-blade saw and multi-layer diamond segments are found in Section 3. Sawing tests and measurement of wear and cutting rate are summarised in Section 4. Section 5 consists of the Statistical machine learning methods of the study such as correlation, PCA, variable importance using random forest regression, and Multiple linear regression using the best subset selection method. The result and detailed discussion of the study and the importance of the study in the future is discussed in Section 6. The key conclusions from the research and their implications are presented in Section 7.

## Rock properties of granite

Nine different types of granite (S1, S2,……, S9) blocks were selected as the workpiece materials for the sawing experiments. The selected granite varieties have substantial market potential, variation in grain size, different shades, and aesthetic properties. Smaller rock samples extracted from the sawn stone blocks were tested in the laboratory for the quantitative determination of rock properties in accordance with the standard test methods suggested by ISRM or other test standards.

Uniaxial compressive strength ($UCS$), modulus of elasticity ($E$), Poisson’s ratio ($\nu$), Brazilian tensile strength ($BTS$), water absorption by weight ($WA$), bulk density ($\rho$), point load strength index ($PLSI$), Cerchar abrasivity index ($CAI$), p-wave velocity (${V}_{p}$) and s-wave velocity (${V}_{s}$) tests were carried out on intact rock samples in accordance with the standard methods suggested by ISRM^43,44^. Protodyakonov strength index ($PI$), Cone indenter hardness ($CI$), Swedish brittleness index (${S}_{20}$), and Cerchar hardness index ($CHI$) tests were carried out as per the methods suggested by the standard tests methods^45–48^. The results obtained from the laboratory test of physico-mechanical rock properties and sawing tests of cutting performance are presented in Table 2.

**Table 2:** Physico-mechanical rock properties.

	S1	S2	S3	S4	S5	S6	S7	S8	S9
$WA$ (%)	0.12	0.06	0.22	0.08	0.09	0.44	0.10	0.11	0.08
$\rho$ (gm/cm^3^)	2.58	2.58	2.58	2.60	2.61	2.57	2.57	2.62	2.61
${V}_{p}$ (km/s)	4.87	5.80	5.69	5.67	5.43	4.25	4.63	4.33	5.73
${V}_{s}$ (km/s)	3.93	2.38	1.42	3.23	1.54	1.24	1.97	1.52	2.13
$UCS$ (MPa)	169.80	161.43	191.52	139.65	118.14	96.48	158.27	138.14	163.89
$E$ (GPa)	51.45	68.15	65.43	57.75	50.75	35.67	53.45	45.97	57.58
$\nu$	0.14	0.23	0.20	0.27	0.11	0.12	0.29	0.22	0.20
$BTS$ (MPa)	9.77	10.62	13.42	7.87	7.61	5.87	8.07	7.58	9.45
$PLSI$ (MPa)	9.60	12.22	12.51	8.74	7.63	4.52	6.94	6.83	9.98
$PI$	4.42	4.39	6.05	3.89	3.80	2.97	5.33	3.60	5.39
$CHI$	36.0	64.0	68.0	46.0	42.0	40.0	56.0	30.0	54.0
$CAI$	2.59	3.07	2.74	2.81	2.56	2.10	2.93	2.32	3.32
${S}_{20}$ (%)	41.33	39.64	26.84	50.87	49.46	58.62	40.51	51.79	34.54
$CI$	6.82	10.64	9.79	7.98	7.37	6.43	8.59	7.85	10.28
$CR$ (m^2^/h)	7.39	4.94	6.64	7.98	7.65	6.73	11.11	10.71	6.07
$WR$ (μm/m^2^)	9.619	13.462	11.657	8.831	8.192	5.069	6.548	6.630	12.193

## Multi-blade saw and multi-layer diamond segments

**Figure 1:**
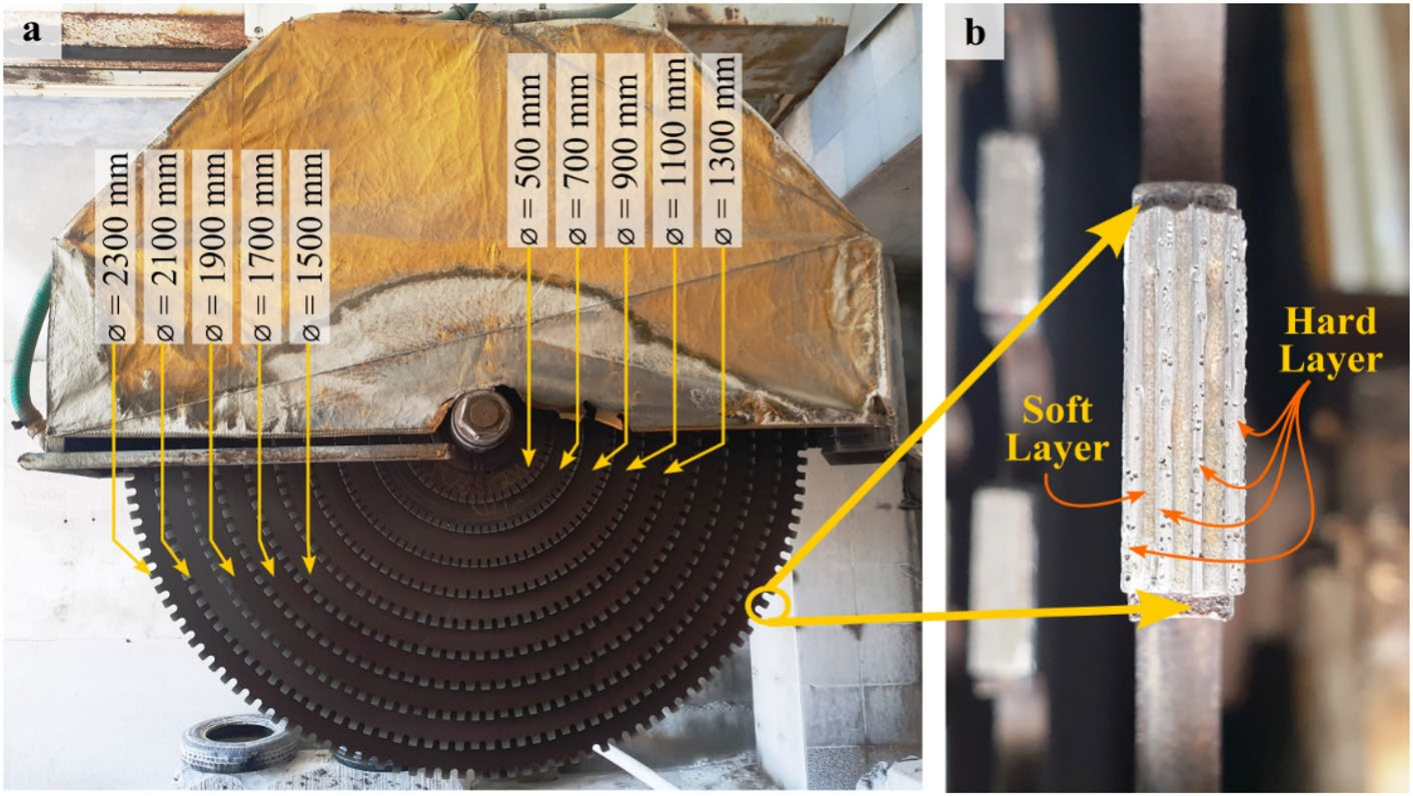
Multi-blade circular saw with (**a**) varying diameters of saw blades (**b**) multi-layer diamond segment brazed over the periphery of the circular saw blade.

A commercial multi-blade circular saw was used in this study for sawing experiments (Fig. 1a). The cutting system of the machine comprised a combination of 10 circular saw blades with the diameter ranging from 500 mm to 2300 mm. The diameter difference between consecutive saw blades was 200 mm. The disc shaped core of each blade had a thickness of 6.5 mm and were made of 75Cr1 grade steel.

The multi-layer segments were brazed over the outer circle of the steel core (Fig. 1b). Linear dimensions of a typical fresh diamond segment were 24 mm ×15 mm ×8.6 mm. The segments were commercially manufactured using the sintering process of powder metallurgy in which the synthetic diamond grits were mixed and bonded together with the help of the Iron (Fe) based metal matrix composites (MMC)^49^. During the segment manufacturing, two different kinds of layers were combined alternatively in it. The primary four layers were the self-sharpening cutting layers comprising diamond particles of 40/50 US mesh uniformly mixed with the metal matrix composite^8^. In between the set of cutting layers, relatively softer layers were introduced comprising iron-based alloys. The diamond grains were not present in the softer layer. The cutting layers actively cut the workpiece surface, while the softer layer provided the free face and facilitated the debris removal from the cutting surface^9^.

**Figure 2:**
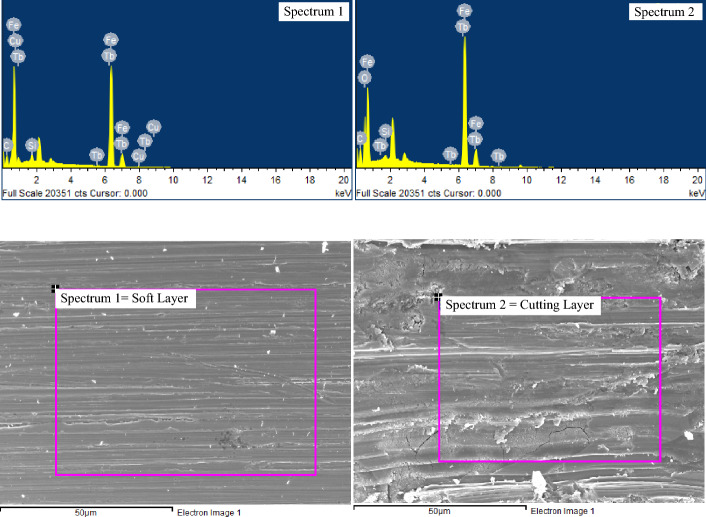
EDS analysis on the surface of a diamond segment with 1024 points and 3 iterations.

To confirm the difference in metallic composition of two adjacent layers of diamond segments, Energy Dispersive X-Ray (EDS/EDX) analysis was performed using an EDS analyzer (Oxford Instruments). Different areas, as displayed in Fig. 2 (Spectrum 1 and 2), were focused during the elemental analysis, and the corresponding elemental peaks were obtained. Different proportions of Fe and Cu can be seen in both spectrums. In spectrum 1, the quantity of Fe and Cu were 66.76 and 3.28, respectively, while in spectrum 2, the values were (23.44, 36.72 measured in weight % for Fe and Cu, respectively). Details of the two EDS spectra of the multi-layer diamond segment measured in weight % and atomic % are listed in Table 3.

**Table 3:** Elemental analysis of diamond segment metal matrix using Energy Dispersive X-Ray (EDS).

Element	Softer layer without diamond grains		Cutting layer with diamond grains	
	Weight (%)	Atomic (%)	Weight (%)	Atomic (%)
Fe	66.76	65.61	23.44	18.57
Tb	24.45	8.44	11.79	3.28
C	4.72	21.58	8.61	31.71
O	–	–	3.81	10.53
Cu	3.28	2.84	36.72	25.56
Si	0.79	1.54	–	–
Al	–	–	0.50	0.82
Ni	–	–	1.96	1.48
Zn	–	–	10.32	6.99
Sn	–	–	2.84	1.06

Some of the mechanical properties of the multi-blade circular saw are as follows; (i) hardness of tungsten carbide blades of multi-blade circular saw range from 70 to 90 HRC (Rockwell C scale), (ii) its toughness is measured in terms of impact resistance hence this tungsten carbide material has lower impact toughness, ranging from 2 to 10 Joules, and (iii) the corrosion resistance can be quantified using corrosion rate measurements. Such as a corrosion rate of less than 0.1 mm per year in aggressive environments.

In similar way, the strength of a diamond segment is a crucial factor in determining its cutting ability. An excessive level of strength will render the crystal resistant to breakage, but it will also lead to the polishing of abrasive grains during usage, resulting in a decrease in sharpness and a decline in tool performance. Hence, the strength of the diamond segment in this study ranges from 130 to 140 Newtons.

## Sawing tests and measurement of wear and cutting rate

**Figure 3:**
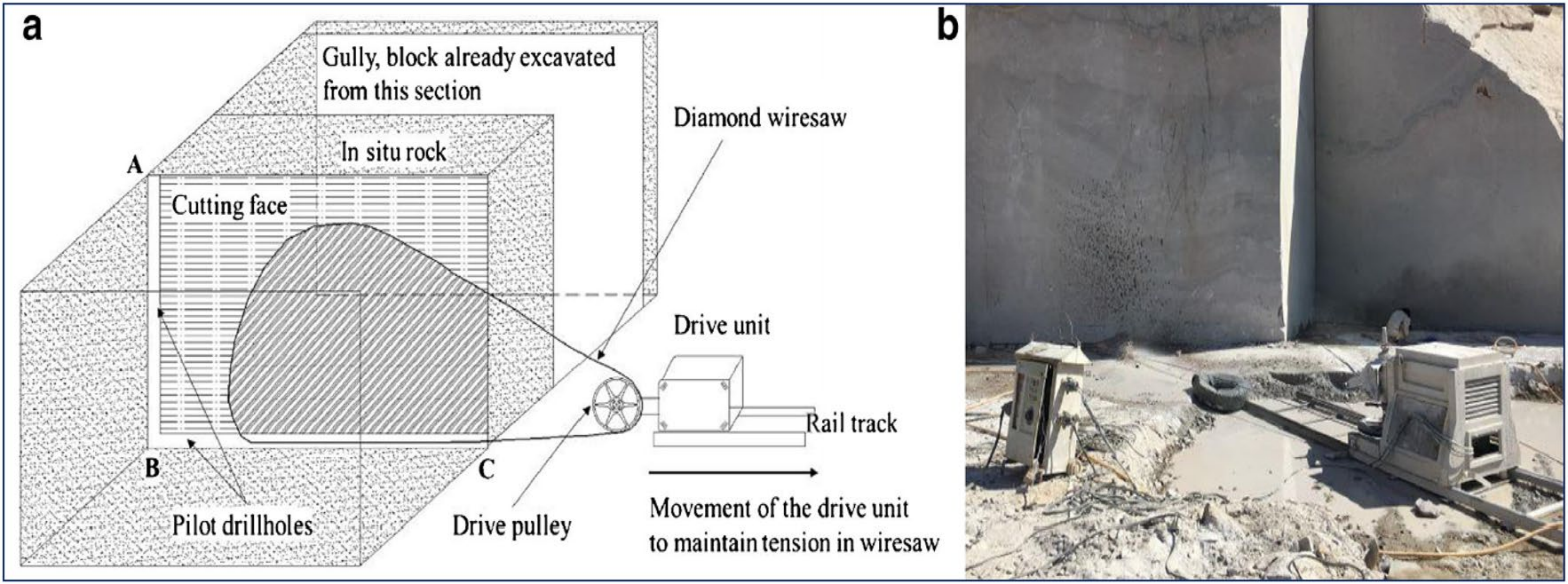
(**a**) Schematic diagram of wire saw cutting in dimension stone quarry; (**b**) diamond wire saw cutting machine used in quarry.

Dimensional stone mining, particularly in granite quarries, involves extracting main rock blocks of 10.6 m× 3 m× 6 m from in situ rock. These basic blocks are subsequently divided into 12 smaller sub-blocks. Block production in quarries involves drilling, pre-split blasting, or diamond wire saw cutting. Diamond wire saw cutting technique uses a continuous loop of 5 mm multi-strand stainless steel rope, ranging from 20 to 80 m in length. Diamond-impregnated beads (10–11 mm) are placed on the wire. The wire has 33–40 beads per meter of running length. The beads are made by sintering or electroplating tiny diamond crystals in a combination of iron powder and other metals. Bead spacing is maintained by inserting springs or plastic spacers between successive beads. Pilot drill holes AB and BC (see Fig. 3) are bored in the in-situ rock, perpendicular to each other.

**Figure 4:**
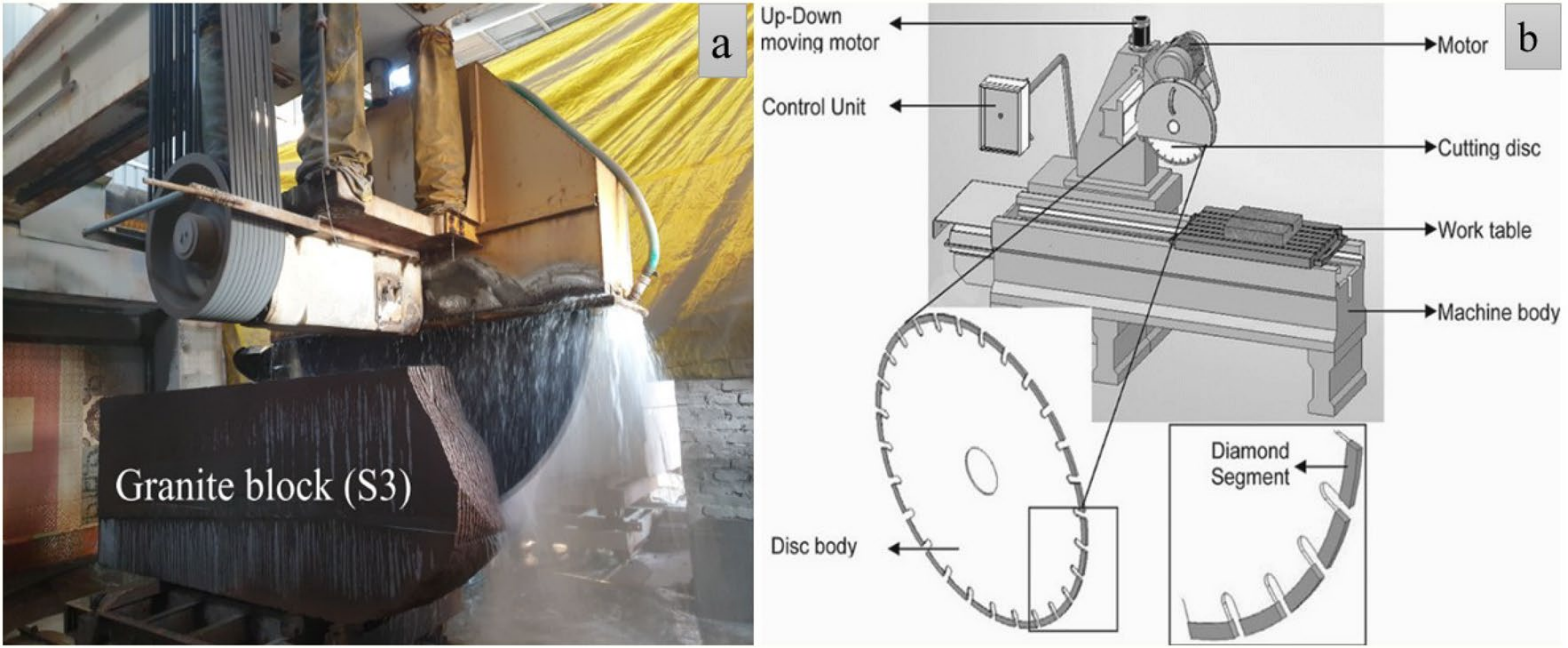
Sawing of granite using multi-blade saw (**a**), and Schematic diagram of sawing machine (**b**).

After the granite block is transported into the processing site sawing tests were carried out on the large-sized granite blocks as shown in Fig. 4**,** It have an average linear dimension of slab area 3000 mm×1000 mm×1250 mm. The block weight was within the range of 25 tones–30 tones. In general, slab with surface area 3000 mm×1000 mm, and 17 mm thickness was sawn using the combination of saw blades in the alternate up-cutting and down-cutting mode.

The saw blades were operated using a 65 kW main electric motor. The machine consisted main bridge supported by four columns; on that, the forward and backward reciprocating movement of saw blades was possible. The feed speed was in the range of50–100 mm/s, the sawing depth was 20 mm–25 mm per pass, and the main machine spindle's rotation speed was kept constant at 343 revolutions per minute. Depth of cut was 20–25 mm for each pass, and it was equal for both up cutting and down cutting pass.

The criteria for evaluation of sawing performance for this study were cutting rate (CR) of granite blocks and wear rate (WR) of diamond segments. Cutting rate was measured as the total areal production of slabs in unit time for cutting of slabs (${m}^{2}/h$). Total area (${m}^{2}$) of the slab surface was measured by multiplying the length ($m$) and width ($m$) of the slabs^12,13,50^.

Wear rate was defined as the ratio of macroscopic linear wear progression (reduction of the height of diamond segment) to the sawn area ($\mu m/{m}^{2}$)^4,20^. From each blade, four number diamond segments were identified for wear measurement and marked using spray paint. Radial wear of marked segments was determined by taking measurements of its height using a digital micrometre before and after the completion of sawing of granite block. Arithmetic mean values of the wear of the worn-out segments were considered for statistical data analysis^51^.

## Statistical machine learning methods

For the purpose of data analysis, RStudio^®^ open-source data analytics software package was used in this study (R version = R− 4.0.2, OS = Windows10 Home 64 Bit)^52^.

### Correlation

**Figure 5:**
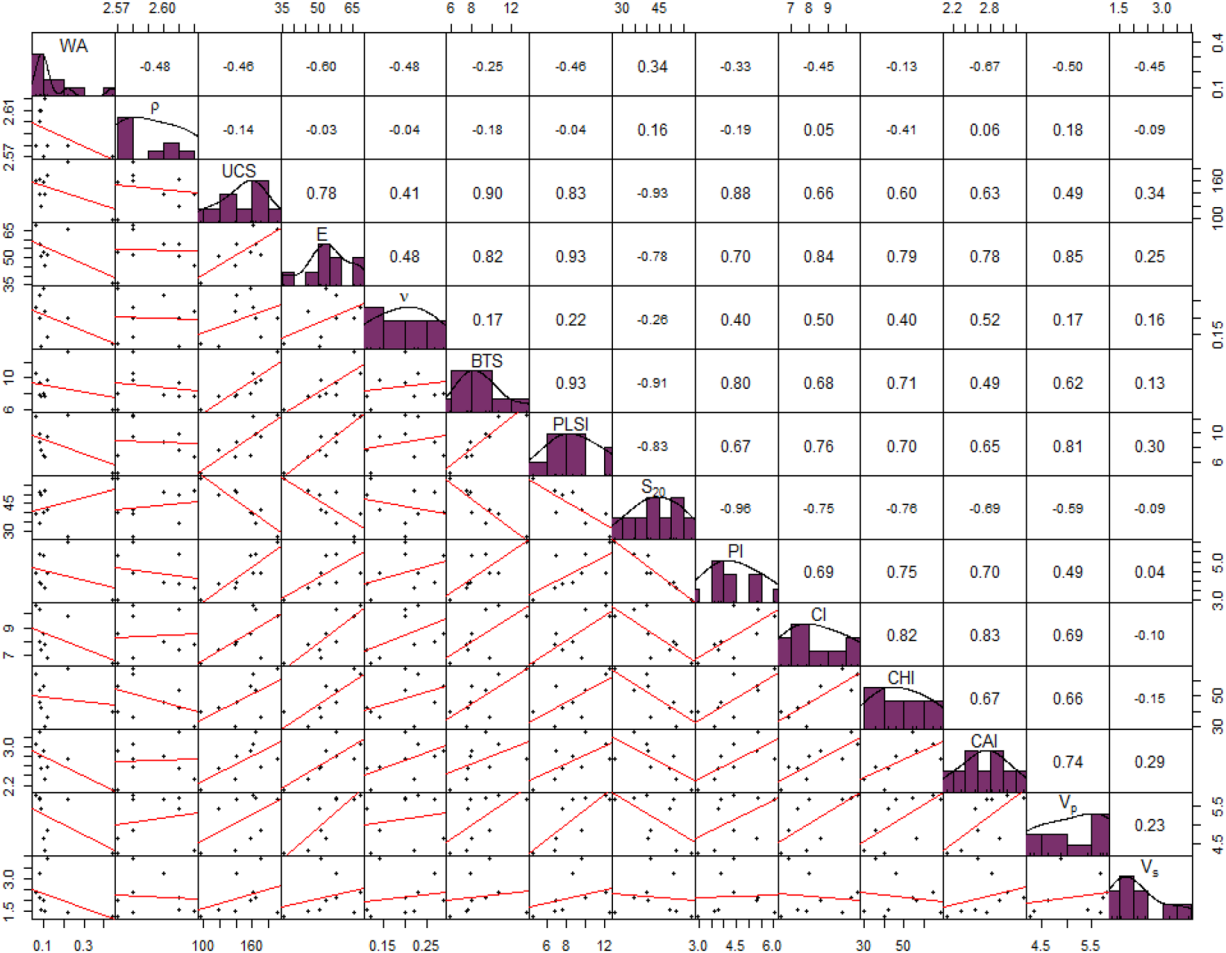
Scatterplot matrix of rock properties.

In order to examine the univariate data distribution and evaluate the pairwise correlation, a scatterplot matrix was generate as presented in Fig. 5, with rock properties as input data^53^. Correlation coefficients ($$R$$) of strength properties revealed the strong positive pairwise correlation ($${R}_{UCS,BTS}= 0.90, {R}_{BTS,PLSI}=0.93, {R}_{UCS, PI}=0.88$$). Similarly, the Swedish brittleness ($${S}_{20}$$) has strong negative correlation with UCS (R = − 0.93). Cone Indenter Hardness and Cerchar Hardness Index exhibit a good positive correlation (R = 0.82). Cerchar abrasivity index is moderately correlated with the Cerchar hardness index (R = 0.67). Elastic property ($$E)$$ has a high correlation with $${V}_{p}$$ (R = 0.85) but relatively weaker correlation of with $$\nu$$ (R = 0.48) and $${V}_{s}$$ (R = 0.25). The rock density ($$\rho$$) and water absorption (WA) showed very weak or almost no correlation with other rock properties.

The linear regression models between cutting rate and rock properties revealed weak univariate linear relationship with almost all rock properties (Table 4). Negative linear relationships of cutting rate with p-wave velocity (${R}^{2}=0.39$), point load strength index (${R}^{2}=0.31$) and Cerchar hardness index (${R}^{2}=0.31$) were exhibited in the linear models. No statistically significant linear relationship has been observed between cutting rate and uniaxial compressive strength, density, water absorption, brittleness, and s-wave velocity.

**Table 4:** Simple linear regression to estimate the cutting rate.

Linear regression model	Standard error	t-value	p-value	F-stat	${{\varvec{R}}}^{2}$
$CR= - 2.531 WA+8.057$	2.154	− 0.399	0.072	0.159	0.02
$CR=18.030 \rho -39.030$	2.147	0.451	0.665	0.204	0.03
$CR= - 0.009 UCS+9.068$	2.159	− 0.349	0.737	0.122	0.02
$CR= - 0.0816 E+12.098$	2.002	− 1.135	0.294	1.288	0.16
$CR=12.499 \nu +5.219$	2.005	1.121	0.299	1.257	0.15
$CR= - 0.381 BTS+11.092$	1.984	− 1.197	0.270	1.433	0.17
$CR= - 0.433 PLSI+11.493$	1.811	− 1.766	0.121	3.120	0.31
$CR=0.059 {S}_{20}+5.119$	2.088	0.783	0.459	0.613	0.08
$CR= - .165 PI+8.424$	2.171	− 0.214	0.837	0.046	0.01
$CR= - 0.510 CI+11.979$	2.015	− 1.086	0.314	1.179	0.15
$CR= - 0.068 CHI+11.007$	1.964	− 1.270	0.245	1.612	0.19
$CR= - 1.585 CAI+11.996$	2.083	− 0.806	0.447	0.650	0.08
$CR= - 2.000 {V}_{p}+18.004$	1.702	− 2.112	0.073	4.461	0.39
$CR= - 0.256 {V}_{s}+8.241$	2.164	− 0.302	0.771	0.091	0.01
$CR= - 0.494 WR+12.206$	1.571	− 2.539	0.039	6.448	0.48

The coefficient of determination (${R}^{2}$) of linear regression for rock properties with wear rate is found to be comparatively higher than the cutting rate (Table 5). For the tested granite rocks, the point load strength index (${R}^{2}=0.90$), modulus of elasticity (${R}^{2}=0.77$), and p-wave velocity (${R}^{2}=0.73$) have strong positive linear relationships with wear rate. Moderately good relations have been observed with cone indenter hardness (${R}^{2}=0.66$), rock brittleness (${R}^{2}=0.57$), and Cerchar abrasivity index (${R}^{2}=0.56$). Comparatively weaker or statistically non-significant relationships were observed between wear rate and density, porosity, and s-wave velocity.

**Table 5:** Simple linear regression to estimate the wear rate.

Linear regression model	Standard error	t-value	p-value	F-stat	${R}^{2}$
$WR=- 11.398 WA+10.780$	2.677	− 1.445	0.192	2.088	0.23
$WR=8.258 \rho - 12.263$	3.046	0.146	0.888	0.021	0.01
$WR=0.070 UCS- 1.315$	2.152	2.658	0.033	7.063	0.50
$WR=0.255 E- 4.641$	1.450	4.896	0.002	23.97	0.77
$WR= 6.388 \nu +7.870$	3.020	0.381	0.715	0.115	0.02
$WR=1.038 BTS- 0.121$	1.828	3.536	0.001	12.51	0.64
$WR=1.035 PLSI+0.053$	0.9828	7.775	0.000	60.46	0.90
$WR= - 0.220 {S}_{20}+18.724$	1.999	− 3.050	0.018	9.304	0.57
$WR=1.689 PI+1.656$	2.470	1.918	0.097	3.68	0.34
$WR=1.528 CI- 3.732$	1.776	3.695	0.008	136.65	0.66
$WR=0.147 CHI+1.996$	2.279	2.354	0.051	5.543	0.44
$WR=5.741 CAI- 6.455$	2.006	3.030	0.019	9.182	0.56
$WR=3.854 {V}_{p}- 10.736$	1.566	4.423	0.003	19.56	0.73
$WR=0.908 {V}_{s}+7.181$	2.922	0.795	0.453	0.632	0.08
$WR=- 0.970 CR+16.594$	2.201	− 2.539	0.039	6.448	0.48

### Principal component analysis (PCA)

Principal component analysis (PCA) was performed on the dataset of 14 rock properties. The PCA orthogonally transformed the dataset into nine non-correlated principal components (PC)^54^. The Eigenvector that exhibited the highest explained variance of the linearly transformed data is termed as the first principal component (PC1)^55^. All the obtained principal components with their respective explained variance are presented in Table 6.

**Table 6:** Percentage of variance of data explained by each principal component.

	PC1	PC2	PC3	PC4	PC5	PC6	PC7	PC8	PC9
Explained variance (%)	58.77	13.84	8.80	7.98	5.47	2.82	1.18	1.13	0.01
Cumulative exp. var.(%)	58.77	72.61	81.41	89.39	94.86	97.68	98.86	99.99	100.00

For the data of 14 rock properties and nine observations, a significant proportion of variance in the data can be explained with PC1 (58.77 %) and PC2 (13.84 %). Moreover, approximately 80% of the variance is explained using the first three principal components, PC1, PC2, and PC3 signifies high range variations in the dataset.

**Figure 6:**
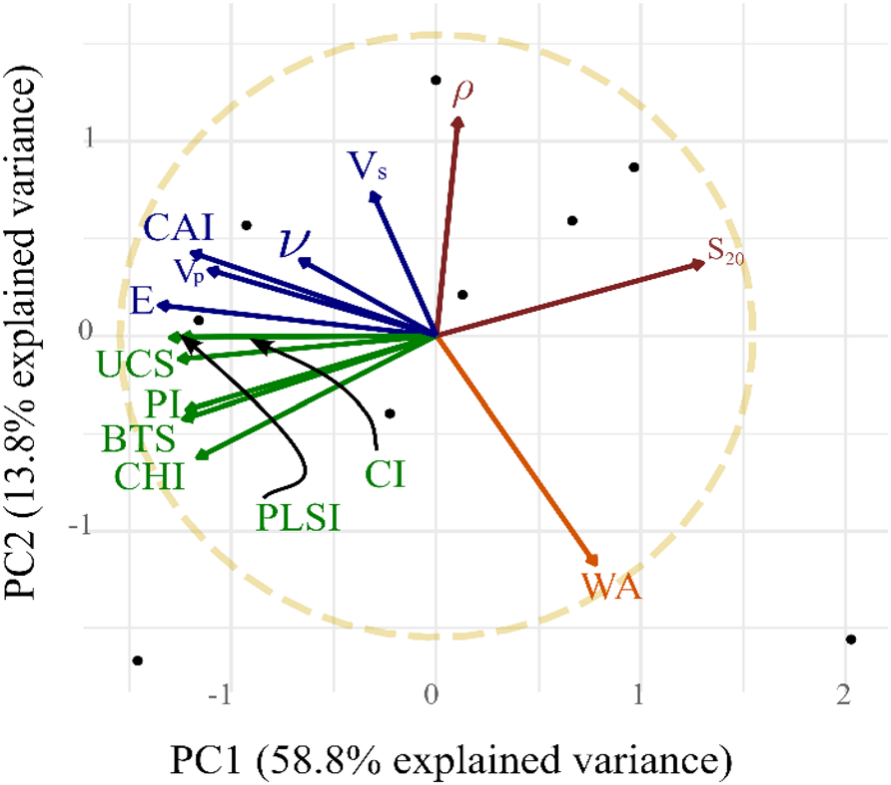
Principal Component Analysis of the rock properties data.

The biplot illustrated in Fig. 6, denotes the explained variance in the data captured by two principal components, PC1, and PC2. The orientation of Eigenvectors of the rock properties indicates that the strength properties such as UCS, BTS, and PLSI are in the same quadrant and exhibit stronger correlations. Cerchar abrasivity index, modulus of elasticity, and p—Wave velocity are present in the same quadrant having significant correlations. Water absorption, Poisson’s ratio, density, and s—Wave velocity are exhibiting weak correlation with other rock properties.

The correlation matrix, PCA, and linear regression suggest collinearity in the data of rock properties. Along with collinearity, it is also observed that any single input rock parameter is not sufficient to estimate the cutting and wear rate of the diamond circular saw. Therefore, the multiple linear regression method was applied to the data to investigate the combined effect of input rock parameters.

### Variable importance using random forest regression

Random forest (RF) regression is a nonlinear and non-parametric method of supervised machine learning suitable for quantitative variables^56^. The cutting rate and wear rate are assigned as dependent variables for the random forest (RF) regression model, and all the 14 rock properties are considered as independent input variables. The main aim of the development of regression models is to investigate the variable importance of rock properties in the model rather than the accuracy of the prediction models.

**Figure 7:**
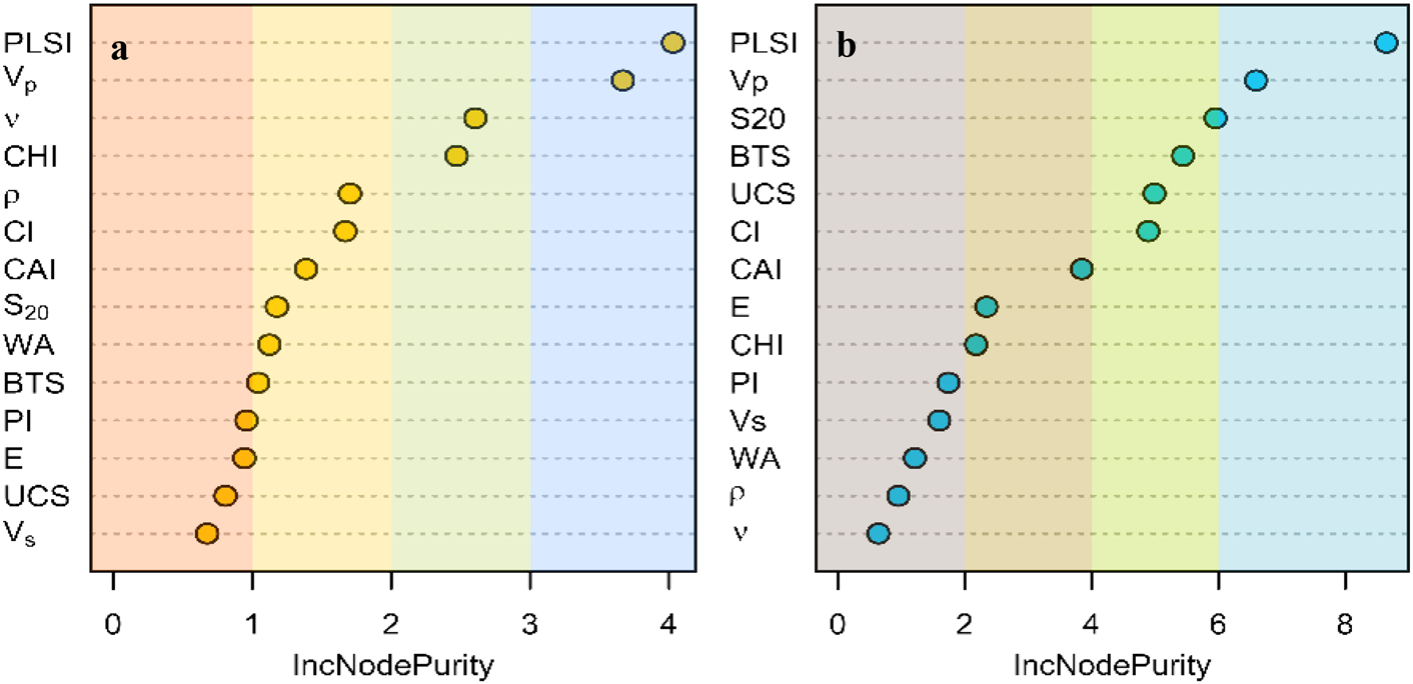
(**a**) Variable importance for cutting rate (**b**) variable importance for wear rate.

The variable importance (Fig. 7) of rock properties in the regression models to estimate the cutting rate and wear rate was evaluated based on the node purity (nodeIncPurity) of the regression trees. The variable with a high value of nodeIncPurity is considered more important than other variables in the model. For regression models, the node purity is calculated based on the residual sum of squares (RSS) before and after the split of the tree on that particular variable^53,57^.

### Multiple linear regression using the best subset selection method

The multiple linear regression (MLR) comprises two or more independent (Eq.1) rock parameters that explain variations of a dependent variable, in this case cutting rate and wear rate^58^.

The multiple linear regression model equation looks like this:

1 $$Y = \beta_{0} + \beta_{1} X_{1} + \beta_{2} X_{2} + \ldots + \beta_{k} X_{k} + \varepsilon$$

where β_0_, β_1_, …, β_n_ are the regression coefficients, X_1_, X_2_, …, X_n_ are the input variables, ϵ are the measurement errors and other discrepancies, and Y is the outcome or predicted variable.

Based on the correlation and PCA outcomes, the nature of data, and the presence of collinearity, the smaller-sized subset regression models were developed^51^. If two similar kinds of independent variables provide similar information, then one variable with lesser significance and low information loss can be omitted in the statistical modelling. Following the same methodology, instead of combining all the 14 input rock properties in a complex linear equation, an exhaustive search algorithm was applied to identify the suitable predictor equation comprising a maximum of 4 numbers of the independent variable. The steps of an exhaustive search algorithm^53^ to determine the best subset methods are described in Table 7.

**Table 7:** Algorithm of best subset selection method for multiple linear regression models.

Step No	Steps of algorithm
1	Let ${M}_{0}$ signifies the null model, having no predictors. The model predicts the sample mean of an independent variable
2	For $k = 1,\dots ,p$ (a) fit all $\left(\genfrac{}{}{0pt}{}{p}{k}\right)$ models that contain exactly $k$ predictors Picked the best four models among these $\left(\genfrac{}{}{0pt}{}{p}{k}\right)$ and called them ${M}_{k1}, \dots , {M}_{k4}$. Here the best model was defined as having the smallest Residual sum of squares, and BIC, equivalently the largest values of Adjusted ${R}^{2}$
3	Selected a single best model from among ${M}_{0}, \dots , {M}_{k4}$ using $BIC or adjusted {R}^{2}$

**Figure 8:**
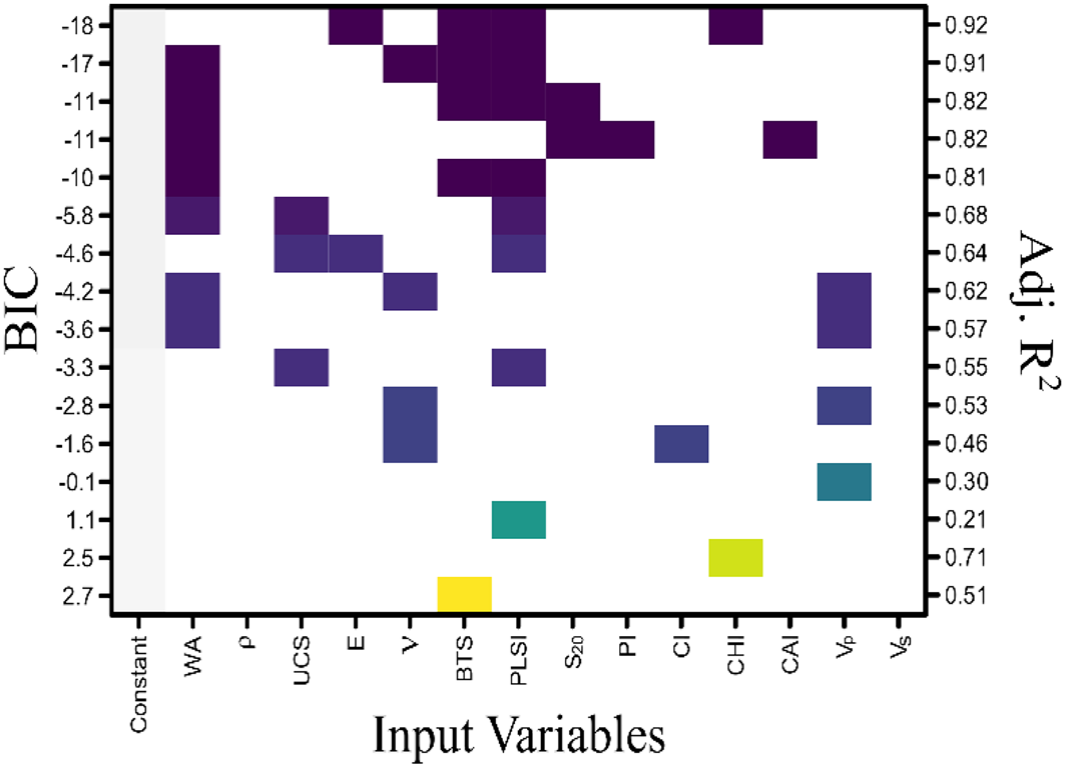
Combination of different input rock parameters for selection of best fit regression model to estimate the CR.

The combination of different regression models to estimate the cutting rate, with their respective values of adjusted R^2^ is given in Fig. 8. It is observed in the tabular graph that among the different combinations of input variables, the predominantly occurring input variables are UCS, BTS, PLSI, and WA. Whereas,${V}_{p}$, Poisson’s ratio, E and CHI are occurring less frequently in the models.

**Figure 9:**
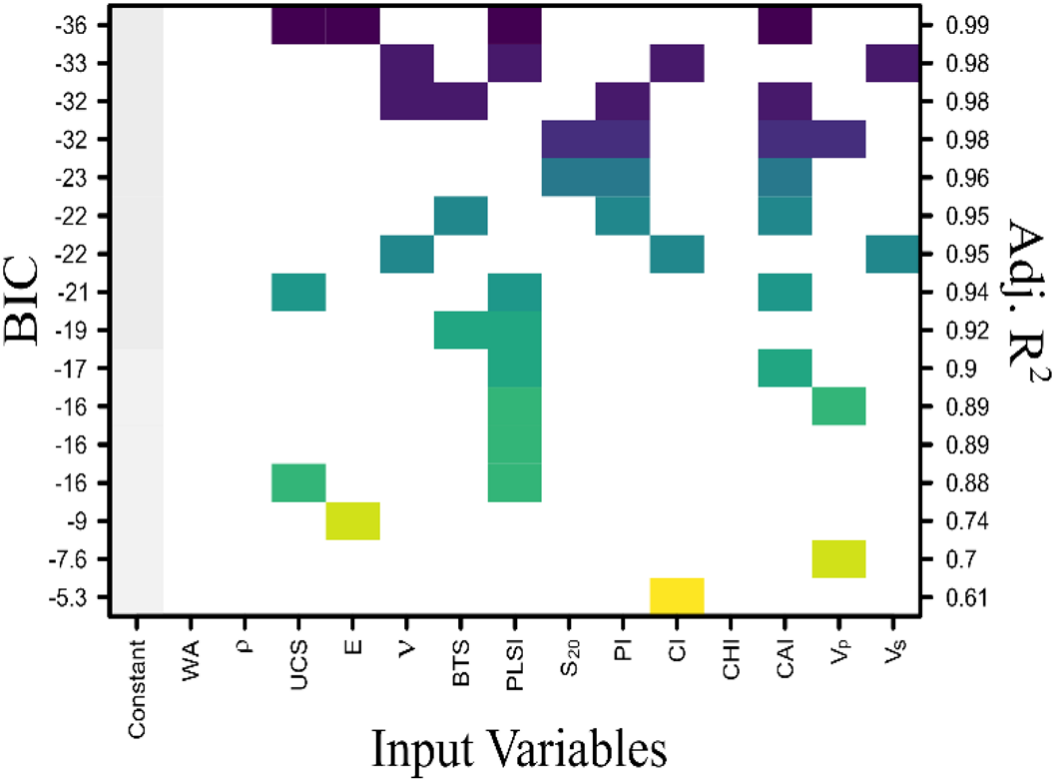
Combination of different input rock parameters for selection of best fit regression model to estimate the WR.

Different combinations of input parameters and their respective adjusted value of the coefficient of determination (adjusted R^2^) for the regression models to estimate the wear rate are presented in Fig. 9. Point load strength index and CAI appear in the models most frequently. UCS, E, Protodyakonov strength index, ${V}_{p}$ and $\nu$ are present in moderation.

## Results and discussion

### Estimation of cutting rate using multiple linear regression model

From the various multiple regression models suggested in Fig. 8 by subset selection algorithm, the model with the largest adjusted coefficient of determination (adj.${R}^{2}=0.92$) and lowest value of Bayesian information criterion (BIC = − 18) in the graphical table was considered as a suitable regression equation to estimate the cutting rate. The model comprised PLSI, E, BTS, and CHI as input variables and cutting rate as a predicted variable. The predictor equation obtained from the multiple linear regression to estimate the cutting rate can be expressed as:

2 $$CR= -3.584 PLSI+0.621 E+2.067 BTS-0.188 CHI-3.691$$

The statistical summary produced in regression analysis (Eq.2) is presented in Table 8. The model is validated using the t-value and p-value of the coefficients. Here, at 95 % confidence level, the coefficient having a p-value less than 0.05 is strongly significant in the model. The p-value for all the coefficients PLSI, E, BTS, and CHI is less than 0.05.

**Table 8:** Summary of the multiple linear regression to estimate the cutting rate**.**

	Coefficient	Std. error	t-value	p-value
Intercept	− 3.691	2.046	− 1.804	0.146
PLSI	− 3.584	0.402	− 8.922	0.001
E	0.621	0.081	7.692	0.002
BTS	2.067	0.301	6.870	0.002
CHI	− 0.188	0.030	− 6.218	0.003
Residual std. error		0.5605		
Degree of freedom		4		
Adjusted R2		0.9243		
F-statistics		25.43		

The analysis of variance (ANOVA) of the regression model to estimate the cutting rate is presented in Table 9. As observed from the correlation and PCA, the strength properties such as PLSI and BTS are correlated, but both are present in the regression model. However, on analyzing the variance explained by each variable using ANOVA, the significance of PLSI, CHI and E are higher than BTS in terms of F-ratio and p-value. As the p-values for PLSI, CHI and E are less than the critical value that is 0.05. Therefore, the major proportion of the variance is explained by one strength, hardness, and elastic rock property in the regression model to estimate the cutting rate. From the two strength variables PLSI and BTS, if one variable (BTS) is dropped from the regression model, then the value of adjusted ${R}^{2}$ reduces to 0.225 and the model becomes returns inaccurate predictions of cutting rate. Therefore, to achieve the higher accuracy of the prediction, all four input variables are kept in the model.

**Table 9:** ANOVA of the multiple linear regression model that estimates the cutting rate.

	Degree of freedom	Sum of square	Mean sum of square	F-value	p-value
PLSI	1	13.847	13.847	44.082	0.003
E	1	5.161	5.161	16.429	0.015
BTS	1	0.793	0.793	2.525	0.187
CHI	1	12.146	12.146	38.668	0.003
Residual	4	1.256	0.314		

**Figure 10:**
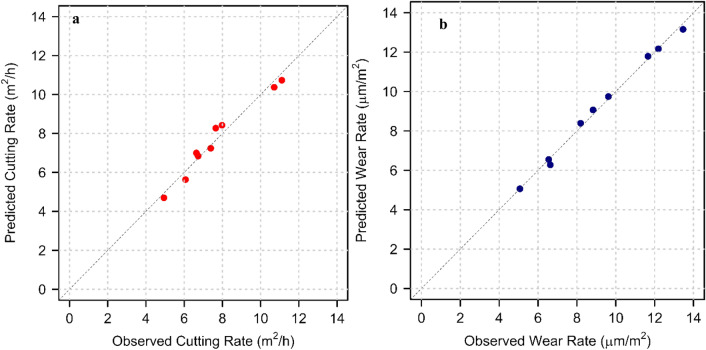
(**a**) Graph between the observed and predicted value of cutting rate (**b**) Graph between the observed and predicted value of wear rate.

The model is validated using the t-value and p-value of the coefficients. Here, at a 95 % confidence level, the coefficient having a p-value less than 0.05 is strongly significant in the model. The p-value for all the coefficients PLSI, E, BTS, and CHI is less than 0.05. The two-dimensional scatterplot between the observed and estimated values of the cutting rate is shown in Fig. 10a, which graphically represents the accuracy of the prediction.

### Estimation of wear rate using multiple linear regression model

The multiple linear regression model to estimate the wear rate was chosen from the graphical table presented in Fig. 8 based on the largest adjusted coefficient of determination $({R}^{2}=0.99)$ and lowest Bayesian information criterion (BIC = − 36) value. The selected model for prediction of wear rate can be expressed in the form of a linear equation as:

3 $$WR=1.699 PLSI+3.703 CAI-0.038 UCS-0.188 E-0.012$$

The statistical summary produced in regression analysis (Eq.3) is presented in Table 10. The p-values for all the coefficients PLSI, CAI, UCS, and CAI have been found to be less than the critical value 0.05.

**Table 10:** Summary of the multiple linear regression model to estimate the wear rate.

	Coefficient	Std. error	t-value	p-value
Intercept	− 0.012	0.916	− 0.013	0.990
PLSI	1.699	0.136	12.510	0.000
CAI	3.703	0.484	7.652	0.002
UCS	− 0.038	0.007	− 5.667	0.005
E	− 0.188	0.038	− 4.954	0.008
Residual std. error		0.2944		
Degree of freedom		4		
Adjusted R^2^		0.9894		
F-statistics		186.90		

The analysis of variance (ANOVA) of the regression model to estimate the wear rate is presented in Table 11. As observed from the correlation and PCA, PLSI and UCS's strength properties are correlated, but both are present in the regression model. The p-values for PLSI, UCS, E, and CHI are less than the critical value that is 0.05. Therefore, the largest proportion of the variance is explained by strength, abrasivity, and elastic rock property in the regression model to estimate the wear rate.

**Table 11:** ANOVA of the multiple linear regression model that estimates the wear rate.

	Degree of freedom	Sum of square	Mean sum of square	F-value	p-value
UCS	1	32.722	32.722	377.452	0.000
E	1	17.774	17.774	205.024	0.000
PLSI	1	9.232	9.232	106.495	0.000
CAI	1	5.076	5.076	58.552	0.001
Residual	4	0.347	0.087		

The p-values for all the coefficients PLSI, E, UCS, and CAI are less than the critical value (0.05). The two-dimensional scatterplot between the observed and estimated values of the wear rate is shown in Fig. 10b, which graphically represents the prediction's accuracy.

### Effect of rock properties on the rock cutting rate and the rate of wear progression

In this context, the relationship between cutting performance and rock properties is likely to be explored to understand how variations in rock characteristics impact the efficiency and effectiveness of the multi-blade circular saw equipped with iron-based multi-layer diamond segments.

The study may analyse factors such as hardness, abrasiveness, and other mechanical properties of different types of rocks to assess how these properties affect the wear of the cutting tool and its overall cutting performance. The goal is likely to identify correlations or patterns between specific rock properties and the tool's wear rates, as well as its ability to cut through different types of rocks.

For example, harder rocks might result in higher wear rates on the tool, affecting its longevity and efficiency. Similarly, the tool's cutting performance may vary based on the abrasive nature of the rocks encountered. Understanding these relationships can be valuable for optimizing the design and application of multi-blade circular saws with iron-based multi-layer diamond segments in various geological contexts.

The investigation of the surface micromorphology of the diamond segments revealed the different modes of wear in the diamond segments: wear of diamond particles and wear of segment matrix^59,60^. The material characteristics of the synthetic diamond differ from the metal matrix composites (MMC)^9,61^. The diamond grain wear occurs during the cutting of rock mainly due to abrasion caused by rock-forming minerals of granite and the cleavage fracture due to impact forces^6,36^. The wear of the metal matrix occurs due to abrasion and erosion^62^. The abrasion of MMC is caused by stream flow of slurry comprising crushed mineral particles, metals chips, and broken diamond grains^11,63^.

The different modes of wear progression in the diamond particles (Fig. 10) are observed in the micrographs: emerging diamond particles, appearance of whole diamond grain, polished and blunt diamond grain, micro fractured diamond grain, macro-fractured grain, and completely broken diamond particle due to cleavage fracture, and pulled out grain^59,64–67^. The area of pulled out grain gets polished due to abrasion and erosion, and new diamond grain emerges somewhere at the cutting surface of the hard layer of diamond segment^36^.

At the scale of 50 $\mu m$, the micromorphology of the diamond segment surface (Fig. 2) shows the dissimilarity in the wear modes on the soft layer and cutting layer. The smooth erosive wear is visible in the soft layer caused by the movement of slurry in a particular direction^62^. Meanwhile, the abrasion due to hard quartz-rich mineral particles is visible in the cutting layer in the form of cavities^6^. The wear track of abrasion-erosion is present in the direction of cutting. Moreover, the micro-fracture reveals the effects of impact loading at the hard layer of the metal matrix^36^.

The statistical results obtained from the data analysis confirm the significant role of rock properties on the wear rate of the diamond segment and the cutting rate of granite. The combined analysis of PCA, correlation, simple and multiple linear regression models, variable importance from random forest regression suggested the most critical parameters influencing the cutting rate are rock strength and deformability, hardness, and abrasivity.

#### Rock strength and deformability

The different strength properties of the granite are investigated in this study, comprising uniaxial compressive strength (UCS), tensile strength (BTS), point load strength index (PLSI), Impact strength in terms of Protodyakonov strength index (PI) in relation to cutting rate and wear rate confirmed comparatively strong correlation with saw performance Each strength property provides separate information regarding the granite; however, these values return nearly similar information for predicting cutting and wear rates. Therefore, the PLSI with the largest correlation value with cutting rate as well as wear rate is found to be the most significant parameters in predicting the cutting rate (p-value = 0.003) and wear rate (p-value ≈ 0.000). The non-parametric regression models developed using RF and the values obtained from variable importance in RF assigned the highest priority to a strength parameter (PLSI). The other strength parameters such as UCS, BTS, and PI were considered relatively less significant in the random forest regression model as they all provided similar information in the model.

In multiple linear regression models, E and BTS properties of granite were found to be significant in the prediction model CR. Similarly, in the prediction models of WR prediction, UCS and E were found to be significant. Along with the strength, the values of Poisson’s ratio ($\nu$) are found to be significant in the RF prediction models of cutting rate. However, on analyzing t-value, F-ratio, and the ANOVA for both regression models, it was observed that their relative significance in the models was less than PLSI, CHI, and CAI. Evidently, the combined analysis of correlation, regression, MLR, and RF indicate the adverse impact of strength and deformability on cutting rate and positive correlation with wear rate.

#### Rock hardness

Two types of hardness were incorporated in this study; (a) Penetration hardness: Cerchar hardness index (b) Indentation hardness: Cone indenter hardness index. From the correlation scatterplot (Fig. 5), the Cerchar Hardness index (CHI) and Cone indenter hardness (CI) are found to be strongly correlated (R = 0.84). In the multiple linear regression model for the prediction of cutting rate, the CHI is found to be a significant parameter (p-value = 0.003). The higher value of CHI along with PLSI is negatively influencing the cutting rate. Observed from the RF regression models, the model with CHI has more inc NodePurity value than other mutually correlated strength properties.

**Figure 11:**
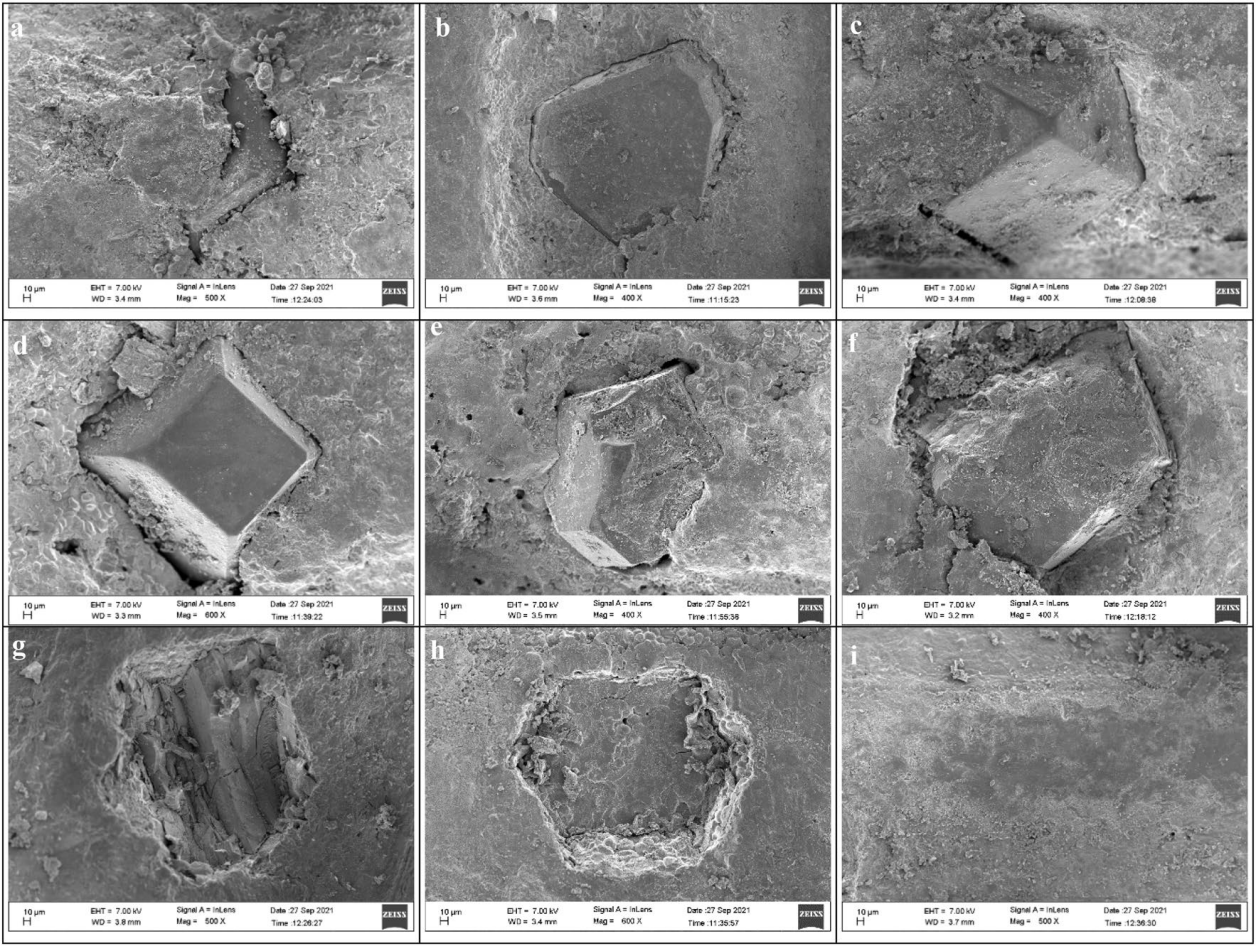
Field Emission Scanning Electron Microscope (FESEM) images of the diamond segments surface highlighting the wear progression in multi-layer diamond segments (**a**, **b**) emerging diamond particle (**c**) fresh diamond particle (**d**) polished diamond particle (**e**) micro-fractured diamond particle (**f**) macro-fractured diamond particle (**g**) completely broken diamond grain (**h**) pull out of diamond particle (**i**) polished surface of metal matrix.

The hardness of granite influences the wearing of diamond grit in the cutting segments. Diamond particles must have higher hardness and toughness than that the mineral particles present in the rock to cut the rock efficiently and smoothly. Granite with higher hardness characteristics tends to resist the cutting^11,68^. In that case, an increase in the normal force in rock cutting damages the diamond grains. Higher hardness of material also increases the rate of polishing on the surface area of diamond grains and ultimately reduces cutting efficiency. In Fig. 11, a fresh diamond grain with sharp cutting edges gets polished rapidly in the case of very hard rocks. Eventually, the cutting efficiency of the segments reduces drastically. To mitigate this problem, the machine operator runs the saw in the dry cutting mode. Due to the friction, the polished layer at the surface of diamond segments gets eroded, and a layer of fresh diamonds appears on the surface. If this method doesn’t work, then a block of abrasive bricks is cut to remove that extra polished layer. Thereafter the granite block is cut.

#### Rock abrasivity

**Figure 12:**
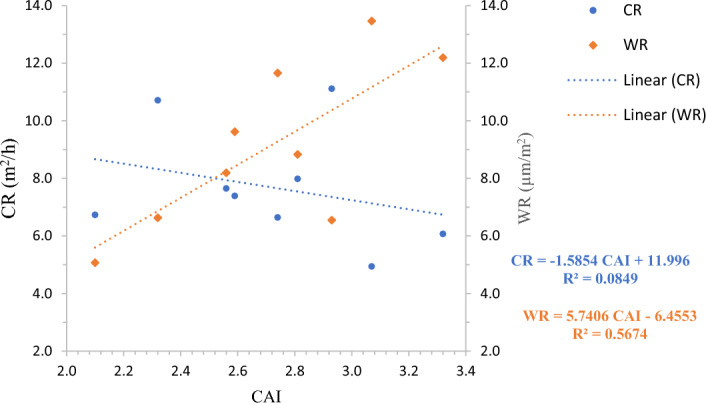
Relationship of abrasivity with (**a**) Cutting rate (**b**) Wear rate.

From the analysis of experimental data of rock properties, cutting rate, and wear rate, it is found that the impact of CAI is more significant in wear rate as compared to the cutting rate. The correlation of CAI with CR is relatively poor ${(R}^{2}=8.49\%),$ whereas a moderately significant correlation is found between CAI and WR ${(R}^{2}=56.47\%)$. The three-axis graph in relation to CAI, CR, and WR reveals the negative impact of CAI on cutting rate and positive correlation with WR (Fig. 12). Similarly, in multiple linear regression to estimate the wear rate of diamond segments, CAI is found to be a strongly significant parameter with nearly a zero p-value (0.001). The variable importance values computed in random forest regression revealed slightly moderate importance in incNodePurity for cutting rate. However, in the RF model for wear rate, CAI has higher variable importance than granite's hardness properties.

The value of CAI of the rock can help understand the role of rock particles in the abrasion of the metal matrix present in the diamond segment^17,36^. A major proportion of the metal matrix is worn out by abrasion and erosion^7^. Therefore, the abrasivity of the metal matrix should be higher than that of rock workpiece materials. High abrasive particles present in the rock forces the erosion of the metal matrix around the diamond particle (wear track around the diamond particle in Fig. 11c).

If the abrasivity of the rock is much lower than the matrix, then during the pullout of the diamond particle (Fig. 11h), the active layer of the metal matrix becomes polished and glazy, reducing the cutting performance^59^. At the same time, the high abrasive minerals inflate the rate of wear of matrix around the diamond grain, ultimately leading to pre-mature pullout of the diamond grit from the active layer of the diamond segment without being used in the sawing. Consequences of that sub-optimal cutting results are high tool wear, higher cost of cutting, and lower tool life^36^. Therefore, to optimize the cutting performance, the erosion of the metal matrix and the wear of diamond particles must be synchronized^64^.

In the process of two and three-body abrasive wear, between the contact surface of the rock, diamond segments, and the mineral particles in the form of slurry, granite with higher CAI tends to exhibit a higher amount of abrasion of the metal matrix. In the sawing of granites composed of high abrasive minerals, wear of the diamond particles occurs on large scale results in its micro or macro fractures. The micro fractures (Fig. 11f) of diamond particles create new cutting edges and assist in cutting. On the contrary, the macro fracture (Fig. 11g) results in a loss of a large proportion of diamond grain, ultimately leading to exhaustion of the diamond particle present on the active layer of segment^69^.

In the view of hardness and abrasivity of the rock workpiece, the granite under this study can be classified into four groups in relation to the selection of diamond segments (a) moderately hard and moderately abrasive granite, (b) hard granite, (c) abrasive granite, and (d) hard and abrasive granite.

**Figure 13:**
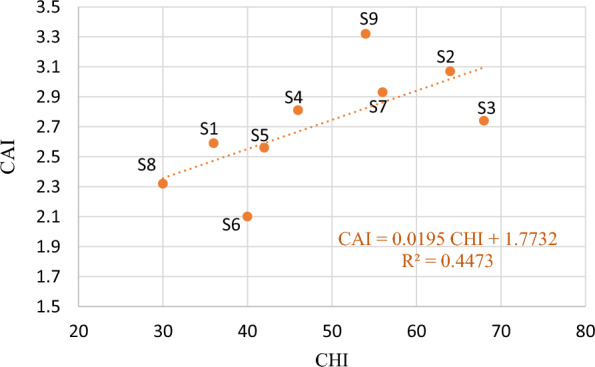
Relation between the values of Cerchar hardness index and Cerchar abrasivity index of granite.

The granite (S8, S6, S1) with moderate hardness and abrasivity can be cut using regular diamond tools (Fig. 13). Granite rocks with high hardness (S7, S3) and moderate abrasivity requires the increased hardness of diamond grains. Similarly, the rock with high abrasivity (S9) and moderate hardness value need high abrasion resistance of metal matrix. Lastly, the rock with significantly large hardness and abrasivity values needs a greater concentration of hard diamond particles with a higher abrasion resistance metal matrix. To obtain the proportionate wear of diamond of and metal matrix, the hardness and abrasivity of the rock should be taken into consideration before the selection of suitable diamond segments.

## Conclusion

The experimental analysis and results reported in this work were meant to evaluate the wear and cutting performance of multi blade combination saw with different diameters having application in natural stone processing. For the tested granite, the following main conclusions were drawn:

1. The rock with higher point load strength index and elastic modulus resulted in lower cutting performance and high wear rates.
2. Cerchar abrasivity index is strongly correlated with wear rate of diamond segments. Rock comprising high concentration of hard abrasive particles rapidly wear out the segment matrix. Similarly, the Cerchar hardness index of granite adversely affects the cutting rate. The granite with higher rock hardness tends to resist the cutting and results in lower diamond grain penetration at the cutting surface of rock workpiece.
3. Rock workpiece materials with similar density may pose the significantly different degree of difficulty of sawing.
4. Surface morphology of the diamond segments reveled the main mode of wear attributed to abrasion, erosion and impact fatigue.

The developed predictive equations to estimate the cutting rate and wear rate were aimed to build the understanding of the combined effect of rock properties on sawing performance. They are valid for the large varieties of granite having rock properties falls under the domain of the predictive linear functions. The equation can be equally useful for diamond tools and saw machine manufacturers, and the stone processing plant engineers.

## Data Availability

All relevant data related to this manuscript are available from the corresponding authors Yewuhalashet Fissha (Email: yowagaye@gmail.com ) , Sohan Singh Rajpurohit (Email: ssrajpurohit@hotmail.com), or Taoufik Najeh(Email: taoufik.najeh@ltu.se ) upon reasonable request.
